# 3D fibre architecture of fibre-reinforced sand

**DOI:** 10.1007/s10035-017-0760-3

**Published:** 2017-09-18

**Authors:** I. Soriano, E. Ibraim, E. Andò, A. Diambra, T. Laurencin, P. Moro, G. Viggiani

**Affiliations:** 1 University Grenoble Alpes, CNRS, Grenoble INP, 3SR, 38000 Grenoble, France; 2 University of Bristol, Bristol, UK; 3 Heriot-Watt University, EH14 4AS Edinburgh, UK; 4 Jacobs UK Ltd., London, UK

**Keywords:** Sand, Fibre, Laboratory, Fabrication, X-ray computed tomography

## Abstract

The mechanical behaviour of fibre-reinforced sands is primarily governed by the three-dimensional fibre architecture within the sand matrix. In laboratory, the normal procedures for sample preparation of fibre-sand mixtures generally produce a distribution of fibre orientations with a preferential bedding orientation, generating strength anisotropy of the composite’s response under loading. While demonstrating the potential application of X-ray tomography to the analysis of fibre-reinforced soils, this paper provides for the first time a direct experimental description of the three-dimensional architecture of the fibres induced by the laboratory sample fabrication method. Miniature fibre reinforced sand samples were produced using two widely used laboratory sample fabrication techniques: the moist tamping and the moist vibration. It is shown that both laboratory fabrication methods create anisotropic fibre orientation with preferential sub-horizontal directions. The fibre orientation distribution does not seem to be affected by the concentration of fibres, at least for the fibre concentrations considered in this study and, for both fabrication methods, the fibre orientation distribution appears to be axisymmetric with respect to the vertical axis of the sample. The X-ray analysis also demonstrates the presence of an increased porosity in the fibre vicinity, which confirms the assumption of the “stolen void ratio” effect adopted in previous constitutive modelling. A fibre orientation distribution function is tested and a combined experimental and analytical method for fibre orientation determination is further validated.

## Introduction

Traditional methods of earth reinforcement for geotechnical systems make use of a large variety of continuous planar synthetic inclusions, usually oriented in a preferred direction, such as strips, fabrics or geotextiles [1]. Multiphase materials provide a high degree of flexibility in design with potential for cost and energy efficiency. An alternative method uses short, flexible fibres as tensile resisting elements, randomly distributed throughout the soil mass. The ability of fibre inclusions to improve the general soil behaviour has been demonstrated in several laboratory experimental studies [2, 3, 4, 5, 6, 7, 8, 9, 10, 11, 12, among others], which identified a multitude of factors that influence the mechanical behaviour of the fibre-reinforced granular soils including stress level, granular soil type and density, fibre type and concentration [13–22]. Particulate analogues based on discrete element modelling (DEM), with parallel treatment of soil particles and fibres, have also been developed to understand how random distributed flexible fibres generate a bond within the soil and affect the kinematics of the particulate matrix [23, 24].

Fibres are most influential when they are evenly distributed throughout the soil and the effectiveness depends on their orientation and the tensile strain distribution resulting from loading [3, 5, 9]. While a homogeneous spatial distribution of fibres is a reasonable practical goal, the distribution of fibre orientations is an outcome which must be known. However, qualitative analysis of fibre orientation at the real geotechnical system scale is missing. Many published studies on laboratory testing of fibre-reinforced soil elements implicitly assume that the fibres are randomly oriented throughout the soil mass. However, indications from recent research show that this assumption may not be appropriate: the fibre orientations resulting from laboratory fabrication techniques are likely to be anisotropic with a preferred horizontal bedding plane [9, 25, 26]. If this pattern is replicated at the scale of real structures, considering that the manifestation of rotation of principal stress or strain axes is present under most types of loading, the consequence of an assumed isotropy of fibre orientation can lead to incorrect predictions of soil design strength parameters. Plausible model simulations of real fibre-reinforced geotechnical structures require comprehensive characterisation of the fibre orientation [17, 27, 28].

Fundamental laboratory characterisation of fibre-reinforced soils and related constitutive modelling need results from tests on homogeneous and reproducible samples. There are many different techniques which have been used for the preparation of samples of granular soils in the laboratory. The soil may be dry, moist or saturated; it may be placed by pluviation through air (or water), spooning or pouring; and may be densified by tamping, tapping or vibration. For the practical use of any experimental finding, it is also highly desirable that the soil elements tested in the laboratory mimic the expected in situ soil fabric [29]. Laboratory samples of fibre-reinforced soils are most commonly prepared using moist tamping (MT), and more recently also by employing a moist vibration (MV) method [30]. Both techniques produce a fibre-reinforced soil fabric that might resemble those obtained respectively in rolled-compacted and vibrated construction fills. Diambra et al. [26] and Ibraim et al. [30] proposed a method to infer the distribution of fibre orientation in cylindrical samples for both MT and MV fabrication methods and for different fibre types using an experimental procedure (cuts through frozen samples) and an analytical model. Although the resulted distributions of fibre orientation corroborate well with laboratory experimental results, a more precise and direct method for description of fibre orientation distribution is still needed.

X-ray computed tomography (CT) has become a very important tool for the three-dimensional characterisation of materials and components, particularly well adapted to soil [31–36]. As this technology continues to advance, high resolution X-ray tomography can provide a practical method for non-destructively acquiring quantitative, three-dimensional microstructural information for particulate soils. This paper seeks to demonstrate the potential application of X-ray tomography to the analysis of fibre-reinforced soils. It therefore intends to develop a fully three-dimensional description of the fibre architecture resulting from normal laboratory sample fabrication processes. The results will also provide a quantitative validation for determination of fibre orientation distribution based on the previous analytical developments [26], including the influence of sample fabrication methods and layering, as well as a local observation of the “stolen void ratio” effect invoked in the continuum modelling of a fibre-reinforced soil [17, 28].

## Experiments

### X-ray CT

A lab-based micro-focus X-ray tomography system was employed to acquire 3D microstructural information to determine the 3D fibre architecture of the fibre-reinforced granular soil. X-ray radiographies of the sample were recorded using a flat panel charge-coupled device (CCD) detector with an exposure time of 0.5 s, turning the sample through 1440 different angles spread through $360^{\circ }$ with a rotation stage. At each angular position, 10 radiographs were averaged together to reduce noise. A 3D field of X-ray attenuation was reconstructed with a modified Feldkamp back-projection reconstruction algorithm as implemented in XAct 2 from RX-Solutions. For this type of composite material, it is essential to know the absorption *versus* intensities in order to use the proper power to achieve maximum contrast between the phases. In the experiments of this study, an electron stream of 90 kV and 110 $\upmu \hbox {A}$ against a tungsten target generated a divergent polychromatic X-ray beam starting from a small focal spot. The divergence of the beam allows geometrical zoom, which in this setup gives a pixel size of 15 $\upmu \hbox {m/px}$ for a cylindrical sample of 20 mm height and 20 mm diameter. However, preservation of small details in the image is important especially for fibre-reinforced composites if automatic image processing is applied and a series of filters and image processing software have been used in order to ensure good contrast quality between the different phases. For the quantitative data analysis, an in-house software tool that tracks each individual fibre was used.

### Samples and scaling of materials’ dimensions

In all X-ray scanning there is an inherent trade-off between the field-of-view and the spatial resolution. Within these constraints, the representativeness of the fibre-reinforced samples consequently becomes one of the key concerns. The efficiency of the fibre treatment is highly dependent on many variables. Among these, the geometrical characteristics (fibre length, fibre diameter and the size of granular particles) form a special set of interrelated parameters. Increasing the fibre aspect ratio (fibre length over fibre diameter) increases the fibre surface area which results in an enhancement of the fibre-matrix interaction efficiency [4, 7, 15, 37]. Maintaining constant the fibre aspect ratio, the fibre reinforcement effect increases with the reduction of the particle size [4, 6, 7, 11]. Michałowski and Čermák [11] suggested that fibre length should be at least one order of magnitude higher than the average grain size, $D_{50}$, to efficiently activate the fibre–grain interaction mechanism. On the other hand, there seems to be an upper limit to the fibre length or fibre aspect ratio beyond which the fibre efficiency remains unchanged [2, 37]. Recent studies by Lirer et al. [38] and Diambra et al. [22] explored in detail the combined effects of fibre and grain dimensions on the efficiency of fibre-reinforced soil composites. Polypropylene short flexible type fibres of 35 mm length with about 0.1 mm circular cross section diameter giving a fibre aspect ratio of 350 and Hostun RF sand with an average particle size diameter, $D_{50}$, of 0.32 mm have been extensively used in the previous research conducted at the University of Bristol, including for the experimental and analytical method of fibre orientation assessment [26]. Samples of 70 mm height and 70 mm in diameter are representative for these fibre dimensions [39]. Considering the sample dimensions employed in this work (20 mm diameter and 20 mm height) and the available active resolution in the X-ray analysis, while preserving sample’s representativeness and direct comparison with the previous studies, some of the fibre dimensional characteristics (fibre length, fibre aspect ratio) had to be scaled down as shown in Table 1.

**Table 1:** Characteristics of fibre, sand and sample used in the previous laboratory work and in this study

	Fibre, sand and sample parameters		Size in pixels$^*$
	Previous studies	This study	
Fibre length, ${l}_{{f}}$ (mm)	35	10	666
Fibre diameter, $d_{f}$ (mm)	0.1	0.1	6
Fibre aspect ratio, $l_f/d_f$	350	100	–
Sand mean size diameter, $D_{50}$ (mm)	0.32	0.32	22
Sample diameter, $D_{sample}$ (mm)	70	20	1333
Sample aspect ratio, $D_{sample}{/}H_{sample}$	1.0	1.0	–
$D_{sample}{/}l_{{f}}$	2	2	–
$D_{sample}{/}D_{50}$	219	63	–
$l_{{f}}{/}D_{50}$	109	31	–
$D_{50}{/}d_{f}$	3.2	3.2	–

$^*$ The size in pixels is based on a resolution of 15 $\upmu$m/px

### Materials

#### Sand

**Fig. 1:**
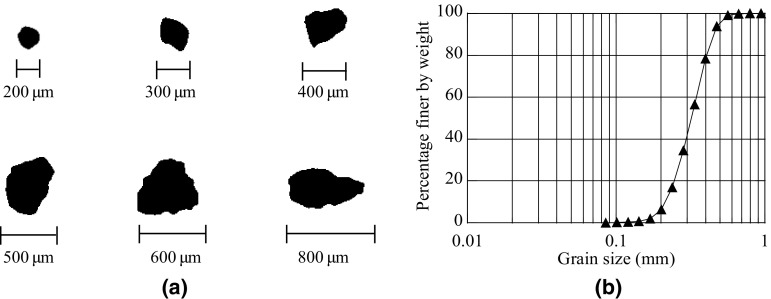
**a** Grain shapes and **b** grain size distribution for Hostun HN31 sand

A single sand type, Hostun HN31 (successor to and identical with the well-studied RF and S28 types), which is a standard European material for laboratory testing has been used for the analysis reported here. This sand of angular to sub-angular grain shape (Fig. 1a) has a silica content $\hbox {SiO}_{2}>98\%$. Its grain size distribution is shown in Fig. 1b and its physical properties are as follows: mean grain size $D_{50}=0.32\,\hbox {mm}$, coefficient of uniformity $C_{u}= D_{60}/D_{10}=1.62$, coefficient of gradation $C_{g}=(D_{30})^{2}/(D_{60} D_{10})=1.0$, specific gravity $G_{s}=2.65$ and minimum and maximum void ratios $e_{min}=0.648$ and $e_{max}=1.041$.

#### Fibres

Polypropylene fibres with a specific gravity of 0.91 have been used in the previous research conducted at the University of Bristol. To facilitate the detection of fibres in the X-ray tomography volumes, and in particular to help distinguish fibres from the water that may partially fill the pores, denser fluorocarbon fibres (PVDF of 1.7 specific gravity, 10 mm length and 0.1 mm diameter) have been selected. A fibre specific gravity of 1.7 is higher than 0.91 of previously employed fibres but still far enough from the 2.65 of the sand. No prior individual mechanical characterisation of this fibre type has been conducted; however, based on the properties provided by the manufacturer, the maximum tensile strength was estimated to be 900 MPa while the tensile modulus of elasticity is about 2.0 GPa.

### Sample preparation

Cylindrical acrylic tubes containing fibre-reinforced samples of 20 mm height and 20 mm diameter were used for fibre orientation determination in the X-ray CT scanning set up. The fibre-reinforced samples were fabricated using either moist tamping (MT) or moist vibration (MV) techniques, following closely the same procedures employed for the samples used in the laboratory element testing of the previous studies [17, 26, 30].

**Fig. 2:**
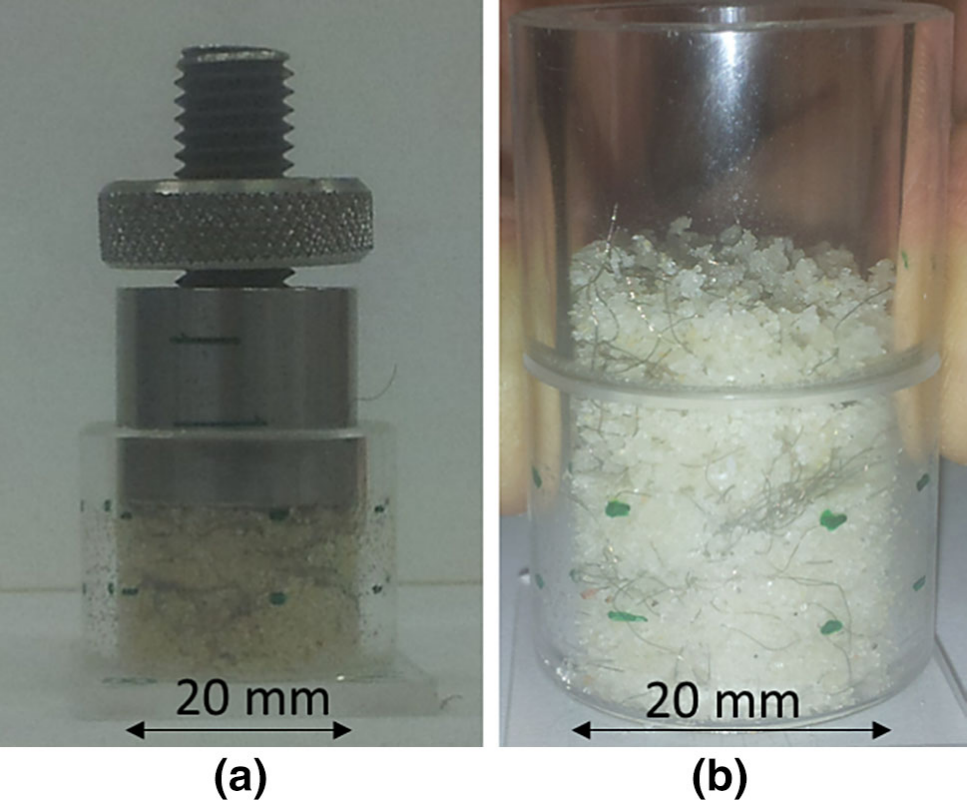
Preparation of samples: **a** MT method, **b** MV method

Both fabrication procedures involve two stages: mixing and formation. The mixing stage is identical for both fabrication methods and begins with the manual mixing of the soil with a controlled amount of water ($W_{w}$). The water is required to enable the mixing of the sand with the fibres and also to prevent fibre-sand segregation. Then, small amounts of fibres are added progressively until, by visual examination, the fibres appear to be well distributed throughout the soil mass. The concentration of fibres ($w_{f}$) used in the reinforced soil samples is defined as the ratio of the weight of fibres ($W_{f}$) and the dry weight of sand ($W_{s}$):

1 $$\begin{aligned} w_f =\frac{W_f }{W_s }\times 100\left( \% \right) \end{aligned}$$

while the moisture content (*w*) is defined in the classical way: $W_{w}/W_{s}$. For both fabrication methods, 10% moisture content was used for the mixing stage [16]. The mixing process is followed by the sample formation: three compacted layers of equal thickness for the MT method and one vibration-densified layer for the MV technique. The compaction of each layer in MT was done manually by a circular tamper of 10 mm diameter, half of cylindrical sample diameter, and finished off by a circular cap of 20 mm diameter (Fig. 2a). For the MV, the full fibre/sand material is carefully transferred avoiding disturbance and fibre/sand segregation into the cylinder acrylic tube (with a cylindrical extension cap) at its loosest density (Fig. 2b) and subjected to a vertical vibration using a shaker with vibration frequency around 55 Hz under a constant surcharge provided by a vertically guided circular top cap. The soil is vibrated until the circular top cap reaches the top of the acrylic tube container.

Four fibre-reinforced samples at two fibre contents ($w_{f}$) 0.25 and 0.50%, have been prepared following the MT and MV procedures, at a fabrication porosity, *n*, around 0.50 ($n =$ Volume of Voids/Total Volume of sample). Table 2 lists the analysed samples. The name of the sample contains the initials of the fabrication method, MT or MV, and the fibre content. Considering the small size of the samples and the very low mass of the constituents, sand and fibres, some variation between the nominal and the real porosity is inherently expected to occur.

**Table 2:** Analysed samples

Fibre content, $w_{f}$ (%)	0.25%	0.50%	Porosity, *n*
Moist tamping (MT)	MT025	MT050	0.5
Moist vibration (MV)	MV025	MV050	0.5

## Results

### X-ray: preliminary scans

**Fig. 3:**
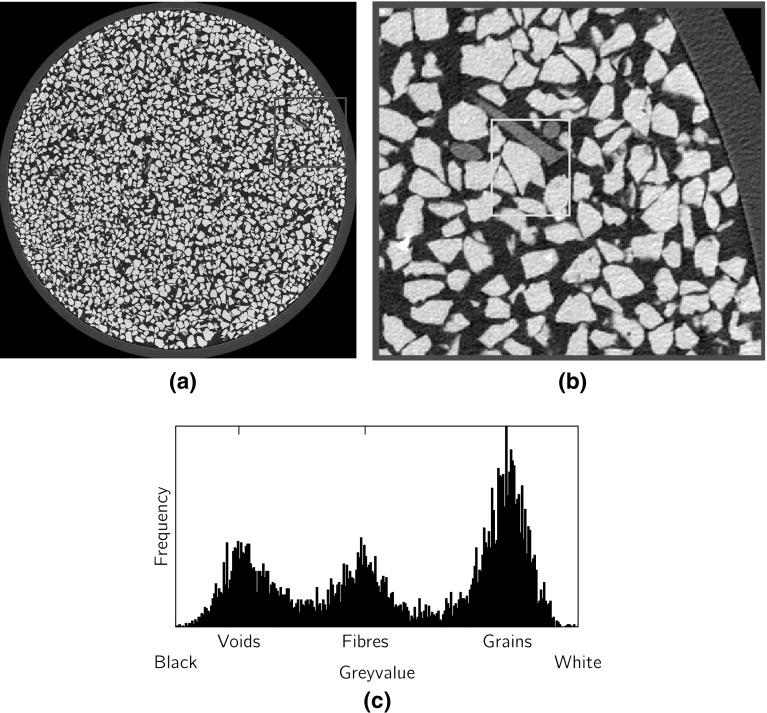
**a** Horizontal cross-section from an X-ray Tomography ($12.5\,\upmu \hbox {m/px}$) of a dry fibre-reinforced Hostun HN31 sand sample, **b** detail containing fibre phase, **c** histogram of the image intensity corresponding to the rectangle detail of (**b**)

Figure 3a presents a horizontal cross-section of a fibre-reinforced sample from a preliminary X-ray tomography aimed at exploring the setting conditions and parameters for the X-ray scanning. This sample was constructed without using any water during the mixing process. The image has a pixel size of 12.50 $\upmu$m/px (acquired with a reduced electron flux of $90\,\upmu \hbox {A}$); fibres appear in the image in a grey colour, clearly distinguished from the sand matrix, which is represented by white grains, and black pores (Fig. 3b). In fact, the intensity histogram relative to its three phases (Fig. 3c) clearly shows the three peaks that correspond to the three phases characterized by different material densities.

The final setting conditions and parameters adopted for the X-ray scanning of all the fibre-reinforced samples are given in the Table 3. The pixel size has been slightly increased to comfortably fit the entire sample in the field of view. The X-ray scanning of each sample has been performed in about three hours; at the end 32 more radiographs spread over $360^{\circ }$ were also acquired allowing any mechanical drift of the imaging system or X-ray source to be corrected. The result of the reconstruction is a 3D volume containing 1400 $\times$ 1400 $\times$ 1400 grey levels, for each sample.

**Table 3:** X-ray scan settings

Source	Detector	Scanning strategy	Geometry
90 kV	Portrait mode	Start/stop (discontinuous rotation)	15 $\upmu \hbox {m/px}$
110 $\upmu \hbox {A}$	2FPS	$+$	
(30 $\upmu \hbox {A}$ for calibration)	Averaging 10 image per angular station	32 Reference images at the end of the scan	
Small spot size	1440 projections		
Plastic filter			

**Fig. 4:**
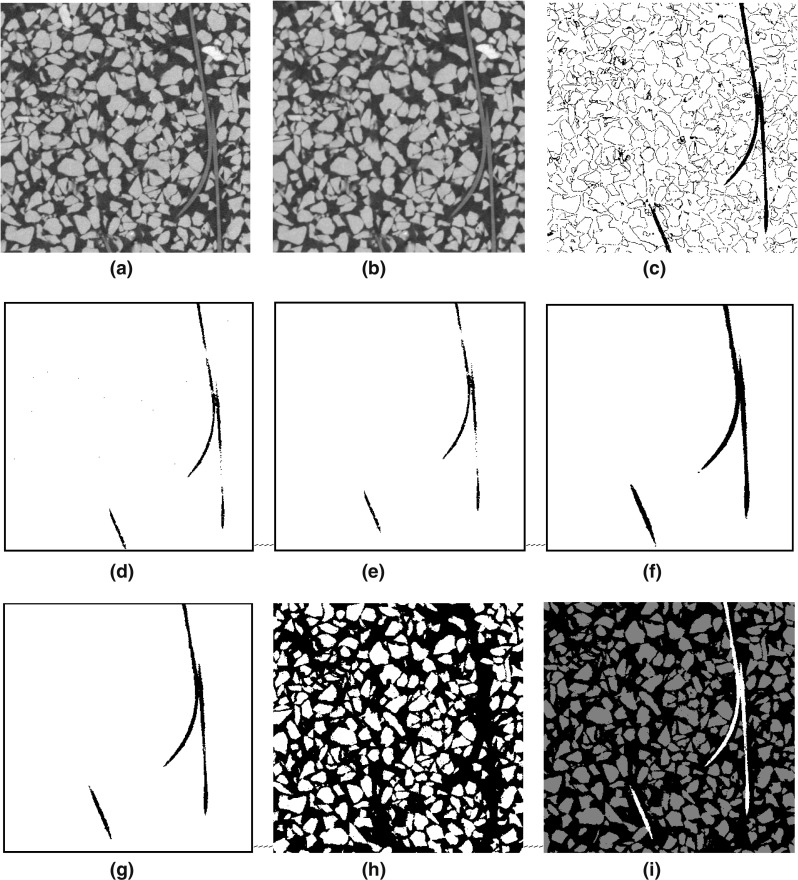
Procedure for obtaining a trinarised image. **a** Original image, **b** step 1—median 3D, **c** step 2—threshold on fibres, **d** step 3—erode, **e** step 4—remove small objects, **f** steps 5 and 6—dilate, **g** step 7—erode, **h** threshold with respect to grains, **i** trinarisation (**g** $+$ **h**)

### Image processing

The micromechanical measurements of the fibre-reinforced samples’ internal structure are obtained by analysing the 3D images obtained from X-ray tomography. The first step is to classify each voxel as belonging to either grain, fibre or pore phases. With these phases defined, a local distribution of porosity will be measured, followed by an assessment of fibre orientation.

In order to proceed to a local, quantitative analysis of the samples, the greyscale images coming from X-ray tomography need to be processed in order to unequivocally assign each pixel to grain, fibre or pore phases. The water phase is also present in the samples to process (10% by dry mass of sand) and its image treatment assigns water voxels to the pore phase (water has a greyscale close to the air phase).

**Fig. 5:**
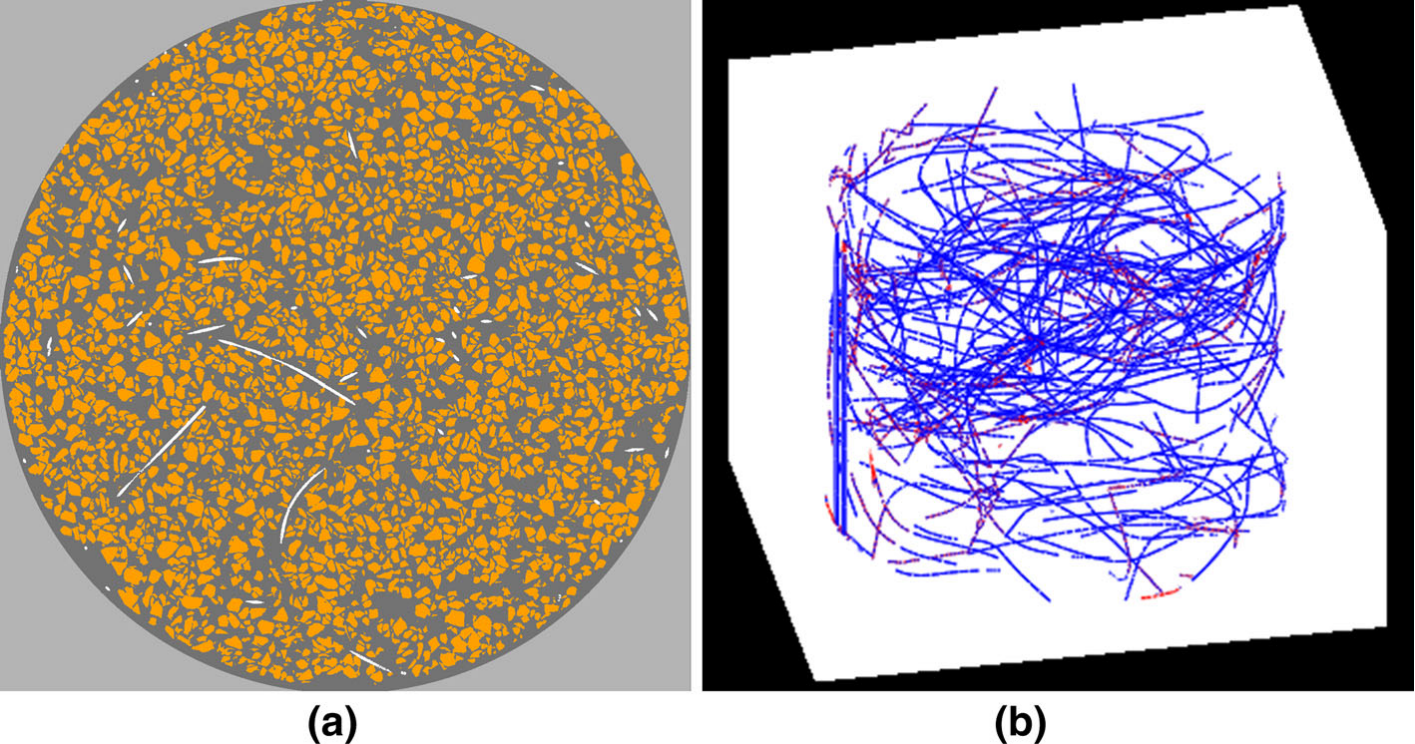
**a** Final output of the trinarised horizontal image, **b** skeletonized fibre network of the MV050 sample

The sequential steps for obtaining a trinarised image are illustrated in Fig. 4. Please note that all image processing operations are done in 3D. The initial image (Fig. 4a) is filtered with a simple 3D median filter or radius 1 to obtain Fig. 4b characterised by smoother grey levels. The more challenging identification of the fibres is described first: in Fig. 4c a threshold is applied (in our case, 22,000–40,000 in our 16 bit smoothed tomography image) to select the fibres (and unfortunately also some pixels on grain/pore edges). These selected pixels are significantly reduced by one cycle of a morphological erosion (Fig. 4d), and the remaining unwanted pixels identified as connected 3D objects smaller than 90 voxels in volume are removed from the picture (Fig. 4e). The fibre map is now dilated—once to recover the initial volume and again to finish filling any voids that may be present in the fibre map (Fig. 4f). Again, with the objective of keeping the correct fibre volume, a final erode step is applied (Fig. 4g) to obtain a final fibre map. The final trinarised image (Fig. 4i) results from superposition of the thresholded grain phase (Fig. 4h) to the fibre map (Fig. 4g). An example of the final output of a trinarised horizontal image of a fibre-reinforced sample (MV050) is given in Fig. 5a. While this procedure directly offers a map of the fibre phase, the quantification of the fibres’ spatial architecture requires an idealised modelling of fibres as 1D objects. To this end, Avizo’s skeletonisation procedure is used and an example of a 3D representation of the entire fibre network of the sample MV050 (fibres rendered with an arbitrary thickness) is shown in Fig. 5b.

### Sample porosity assessment

An in-house python script was developed to make local measurements of sample porosity. For practical reasons, the porosity is preferred over void ratio for the simple reason that it is a ratio of occupancy with respect to a *total* (local) volume which is convenient to set when using 3D images. Concerning the issue of the size of the representative sample sub-volume, this is set by successively measuring the porosity of a cube with an increasing volume size. When the porosity measurements all converge and become stable this is taken as the sub-volume size for the analysis, which in this case represents a cube of $55\times 55\times 55$ pixels (approximately $2.5\times 2.5\times 2.5\,\hbox {D}_{50}$). The mesh of points that has been chosen as a basis for measurements (on which the sub-volumes will be centred) has a spacing of 4 pixels in each direction, which means there is a very significant overlap of the measurement windows. In order to avoid the inherent effects of the sample borders, the porosity has been calculated considering an inner volume of the sample.

**Fig. 6:**
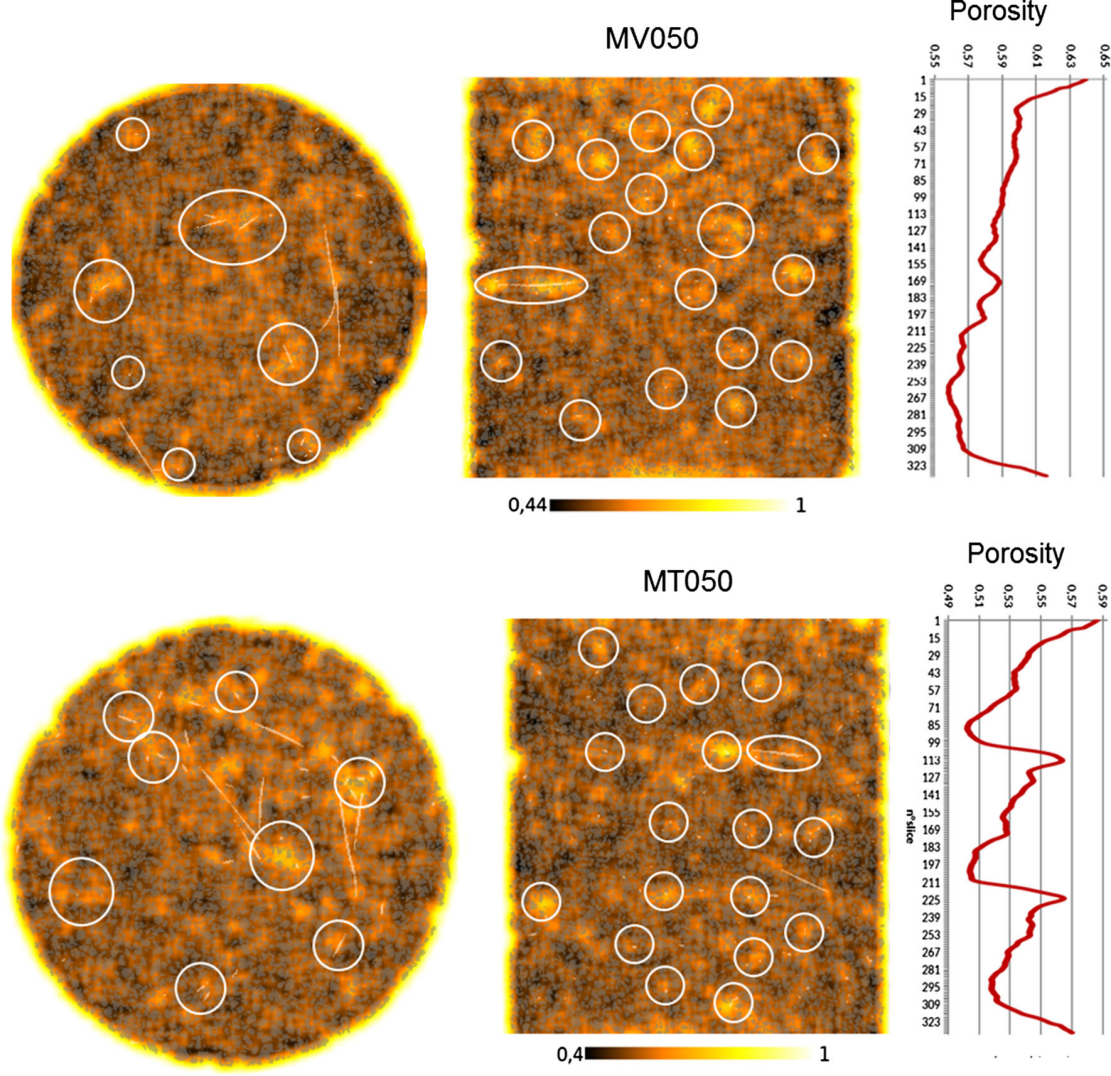
Porosity maps of horizontal and vertical sections and plot of the average porosity along the vertical z-axis for MV050 (top) and MT050 (bottom) samples

Figure 6 shows the porosity maps for horizontal and vertical sections of MV050 and MT050 samples. The results are reported in scale of orange. Lighter colours correspond to higher porosity, while a lower porosity is represented by darker shades. The fibres are also superimposed on horizontal and vertical porosity maps of the selected sections. Figure 6 also shows the variation of the average porosity along the vertical z-axis for MV050 and MT050 samples. For the MV050, the porosity slightly and gradually increases from the bottom up to the top of the sample. The larger values of porosity measured at the bottom of the sample are due to the contact between the grains and the cylinder base and confirm the numerical simulation results of Marketos and Bolton [40]. DEM studies by Huang et al. [41] showed similar trends. For the MT050 sample, the layering effect is explicitly revealed through the existence of three distinct porosity evolutions corresponding to the three layers. Within each layer, the porosity appears lowest at the bottom and higher at the top. However, for both fabrication methods, a superficial layer of higher porosity is present at the top of the samples.

From visual inspection of the selected sections in Fig. 6 (see also Fig. 5a), it seems that the presence of fibres strongly affects the porosity in their vicinity. In most cases, the porosity is higher locally around the fibre inclusion (circles have been added around some fibres to highlight this effect on the sample sections in Fig. 6). This can be related to a possible wall type effect, in which granular particles near a rigid wall boundary show higher porosity. For a granular material, the distance over which the local porosity is affected was estimated to be about 4–5 average particle size diameters from a vertical sample wall [42]. However, for fibre-reinforced soils, De Larrard [43] estimated the perturbed zone from the face of rigid cylindrical fibres mixed with a granular soil (having an average particle size diameter of the same order of magnitude of the fibre diameter) to be much smaller, about 10% of the average particle size.

**Fig. 7:**
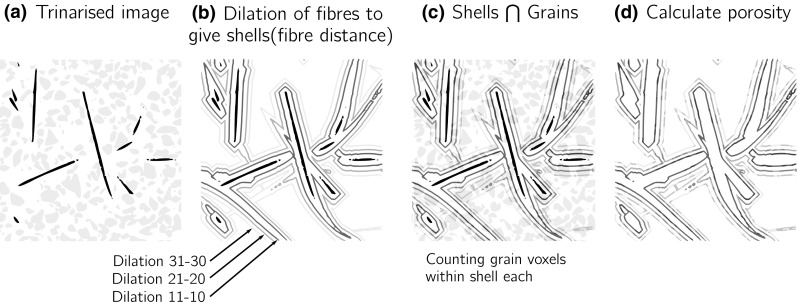
Illustration of global measurement of porosity versus distance from the fibre surface with illustration using shells at 11, 21 and 31 pixels distance from the surface of the fibre. **a** Trinarised image, **b** dilation of fibres to give shells (fibre distance), **c** shells $\bigcap$ grains, **d** calculate porosity

In order to quantify the porosity in the vicinity of the fibres, an additional bespoke image analysis script was written in python to measure the evolution of porosity with increasing distance to each fibre. To achieve this direct calculation, a trinarised image is used (Fig. 7a); for *n* increments of one pixel up to a maximum fixed scanning distance in pixels, the fibre phase is dilated and the volume defined by the previous dilation ($n-1$) is subtracted, leaving a one-pixel thick shell at a distance *n* from the surface of all fibres (Fig. 7b). It is important to note that this global operation naturally handles fibre intersections. Each shell is intersected with the grain phase (Fig. 7c), and thus the porosity is calculated as: (volume of the shell − volume of grains within shell)/(volume of shell) (Fig. 7d).

**Fig. 8:**
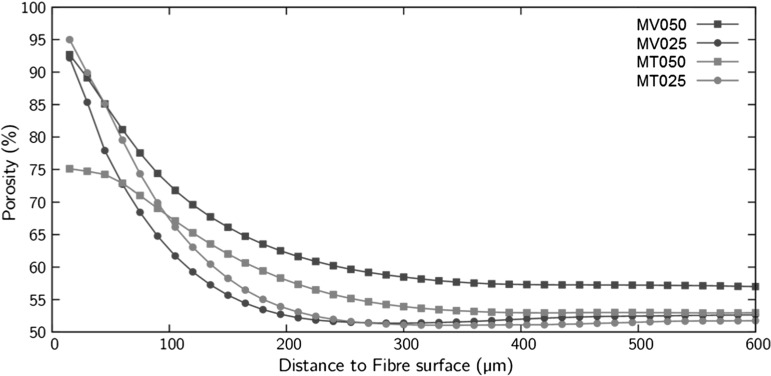
Results of the average porosity around all the fibres from the surface of fibres for all MV and MT fibre-reinforced samples. For reference, fibre diameter is $100\,\upmu \hbox {m}$

The application of this code to all four-scanned fibre-reinforced samples shows that the perturbed porosity distance is about the same order of magnitude of the average particle size, $\approx$300 $\upmu \hbox {m}$, and independent of sample formation method (MT or MV) and fibre content (Fig. 8). These observations confirm a conjecture related to the role of the fibres on the global interaction mechanism by Diambra et al. [17], Diambra et al. [28] and Muir Wood et al. [44], which implies an increased porosity in the fibre vicinity. As a consequence, the porosity of the granular matrix decreases, hence the so-called “stolen void ratio” effect adopted in previous constitutive modelling of fibre-reinforced sand systems.

### Fibre orientation distribution analysis

First, the determination of fibre orientation from the X-ray CT scanning of fibre-reinforced samples is presented. Acknowledging that the use of these techniques cannot be employed on an everyday basis, it is important to test and assess the validity of the previously proposed procedure for the determination of fibre orientation distribution by Diambra et al. [26]. Therefore, comparison of the results from X-ray investigation with the fibre orientation deduced with the procedure proposed by Diambra et al. [26] will follow.

#### X-ray analysis

**Fig. 9:**
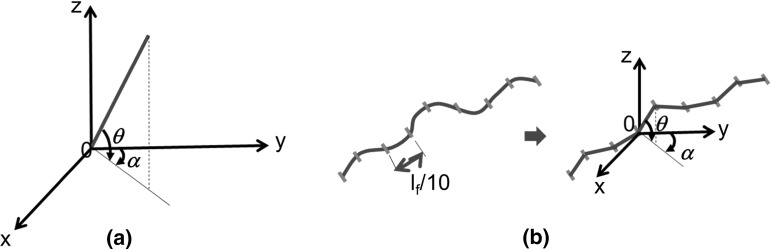
Coordinate system and notations used for detection of fibre orientation: **a** straight fibre, **b** flexible fibre and division into straight segments for the X-ray CT scanning analysis

The spatial orientation of fibres in a composite matrix can be described in different ways. Assuming a straight rigid fibre, one description can be related to the detection of an in-plane angle, $\upalpha$, which is the angle between the fibre projection on the x–y plane and the y axis, and out-of-plane angle $\theta$, which is the angle between the fibre and its projection on the x–y plane (Fig. 9a). In the Fig. 9a, the z-axis coincides with the vertical cylindrical sample’s axis. However, the fluorocarbon type fibres are not straight and the detection of the angles $\theta$ and $\upalpha$ cannot be based on the use of the extremity points of the fibre. Therefore, an automated procedure has been devised, consisting of dividing the fibre into several sub-sections, each one assumed to be straight and in length approximately one tenth of the total fibre length (1 mm). Figure 9b shows one flexible fibre and illustrates the coordinate system and the notations used. The division of the fibre into sub-sections is supported by DEM simulation results of idealised fibre-reinforced granular materials conducted by Ibraim et al. [23] and Maeda and Ibraim [24]. These analyses showed that the tensile stress distribution mobilised along an individual flexible fibre embedded in a loaded mixture is not uniform and is controlled by fibre path orientation, with highest tensile stresses mobilised on those fibre segments oriented towards the minor principal strain direction coinciding with the tensile strain direction.

The 3D skeletonised fibre network architecture reconstructed by the X-ray CT (Fig. 5b) contains all the fibre spatial information. For each fibre-reinforced sample, the 3D spatial information was transferred into an in-house Matlab program and the 1 mm fibre length segments were automatically traced along the fibres. For each segment, a set of ($\theta$, $\upalpha$) angles used to analyse the spatial geometry and orientation of individual fibres was subsequently produced.

**Fig. 10:**
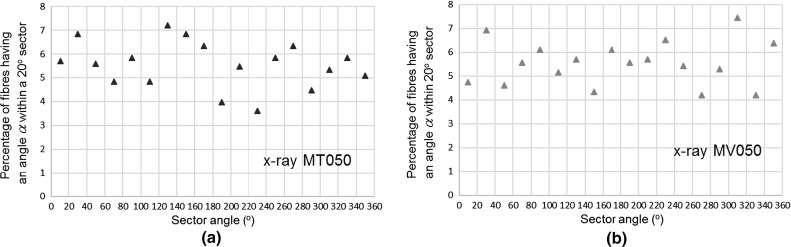
Variation of the percentage of 1 mm fibre segments having an angle $\upalpha$ within a circle sector of $20^{\circ }$ for equal intervals for: **a** MT050 and **b** MV050 samples

In the context of cylindrical soil samples, the axisymmetry of fibre orientation with respect to the vertical axis is an important characteristic of the 3D fibre architecture network. The laboratory fabrication methods are expected to produce an axisymmetric distribution of fibre orientation and the distribution of $\upalpha$ angles provides information on the randomness of in-plane fibre orientation. The variation of the percentage of 1 mm fibre segments having an angle $\upalpha$ within equal circle sector intervals of $20^{\circ }$ for up to $360^{\circ }$ is shown in Fig. 10a, b for MT050 and MV050 samples, respectively. The variation of $\upalpha$ angles for both fabrication methods suggest almost random in-plane distributions of orientations of fibres with a standard deviation below 1% for both fabrication methods. Therefore, in the description of the fibre orientation, the effect of in-plane $\upalpha$ angle can be ignored and the analysis can solely refer to the distribution of $\theta$ angles.

**Fig. 11:**
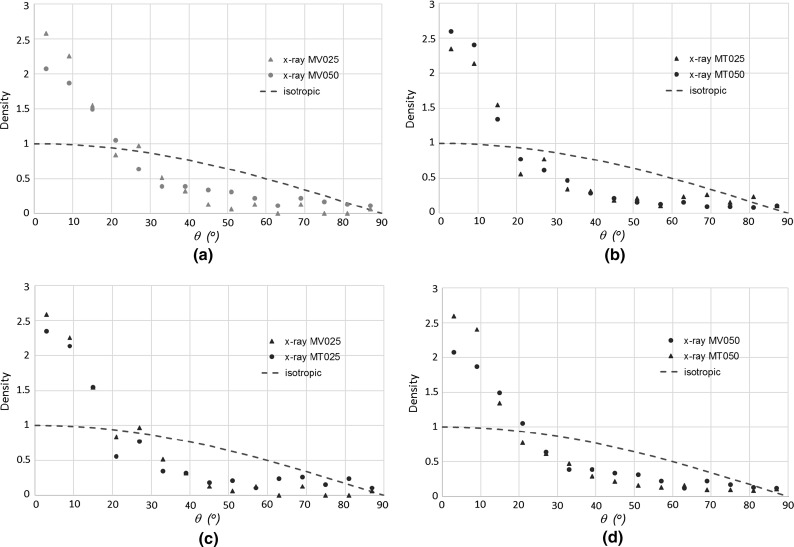
Fibre orientation distribution with respect to the horizontal direction resulted from X-ray analysis: **a** effect of fibre content for MV fabrication method, **b** effect of fibre content for MT fabrication method, **c** comparison between MT and MV fabrication methods for 0.25% fibre content, **d** comparison between MT and MV fabrication methods for 0.50% fibre content

Figure 11 summarizes the fibre orientation distribution based on the measured out-of-plane $\theta$ angle from the X-ray CT images for all the fibre-reinforced samples. The statistical analysis was performed for all $\theta$ directions of the first quadrant between $0^{\circ }$ and $90^{\circ }$ and the density probability of the fibre orientation distribution is calculated based on the total number of fibres oriented within an angle interval $\varDelta \theta = 6^{\circ }$. For each angle interval, 15 in total, the density probability is represented by a point and all occurrences of the angle orientations sum to unity. For comparison, a theoretical density probability function for an isotropic distribution of fibre orientation (dashed lines) is given in each figure. The effect of fibre concentration on the distribution of fibre orientations is presented in Fig. 11a, b for MV and MT fabrication methods, respectively. Overall, for both fabrication methods, the fibre concentration does not seem to have any effect on the fibre orientation, although a very slight difference is detected between MV025 and MV050 samples. This difference is retained when MV050 is compared with MT050 (Fig. 11d) but no differences are observed between the fabrication methods for 0.25% fibre content (Fig. 11c). The sample layering in MT therefore appears to have a very limited effect on the fibre orientation distribution, as similarly claimed by Ibraim et al. [30]. As can be observed, both fabrication methods generate fibre orientations that consistently deviate from the isotropic orientation, with a larger number of fibres oriented within angle intervals close to the horizontal. While for an isotropic fibre distribution about 50% of fibres are oriented between $0^{\circ }$ and $30^{\circ }$, this percentage increases to 81% for MT050, 77% for MT025 and 75% for MV050, 86% for MV025. These results corroborate well with the assessments made without X-ray tomography on larger samples (D $=$ 70 mm and H $=$ 70 mm) formed by the same fabrication procedures by Ibraim et al. [30] using the same sand and flexible fibres of similar diameter.

#### Formulation of fibre orientation distribution and validation

**Fig. 12:**
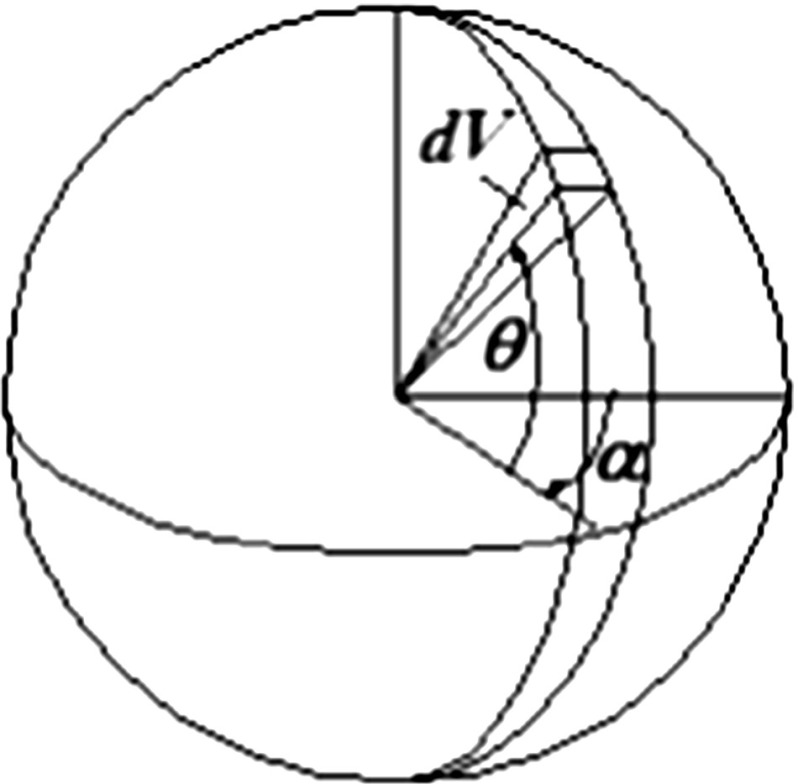
Sphere and coordinates used to define fibre orientation

For short and straight fibres axisymmetric oriented with respect to the horizontal plane, a theoretical expression for the volumetric concentration of fibres, $\rho (\theta )$, in an infinitesimal volume $\hbox {d}V$ having an orientation of angle $\theta$ above the horizontal plane (Fig. 12) can be given by the following function [9]:

2 $$\begin{aligned} \rho \left( \theta \right) =\bar{\rho }(A+B\vert \cos ^n\theta \vert ) \end{aligned}$$

where $\bar{\rho }$ is the average volumetric concentration of the fibres, defined as the total volume of fibres ($V_{f}$) per sample volume (*V*):

3 $$\begin{aligned} \bar{\rho }=V_f/V \end{aligned}$$

and *A*, *B* and *n* are constants linked by the following relation which ensures that the volumetric integral of function (2) over the whole spherical integration domain in Fig. 12 is equal to $\bar{\rho }$:

4 $$\begin{aligned} B=\frac{1-A}{\mathop \int \nolimits _0^{\pi /2} \hbox {cos}^{n+1}\left( \theta \right) d\theta } \end{aligned}$$

The assessment of the fibre orientation distribution requires the calibration of two of the three constants *A*, *B* and *n* specified in (2) [the third one can be evaluated from (4)] and can be conducted based on previous developments proposed by Diambra et al. [26] which involve an experimental procedure and an analytical model.

**Fig. 13:**
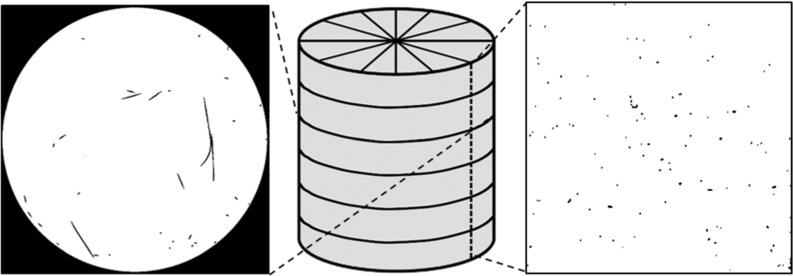
Sections from X-ray tomography through the sample and two examples of horizontal and vertical sections containing the cut fibres from X-ray images

**Table 4:** Results from the determination of fibre orientation distribution based on analytical and experimental procedures [26]

Sample	X-ray experimental determination			Analytical model				
	$N^{V} (\mathrm{fibre/cm}^{2})$	$N^{H}(\mathrm{fibre/cm}^{2})$	$N^{V}/N^{H}$	*n*	*B*	$N^{V}$	$N^{H}$	$N^{V}/N^{H}$
MT050	27.8	11.5	2.44	8	2.461	31.35	12.84	2.44
MV050	30.2	14.0	2.16	6	2.188	31.0	14.2	2.17

The parameter *A* is set equal to zero

**Fig. 14:**
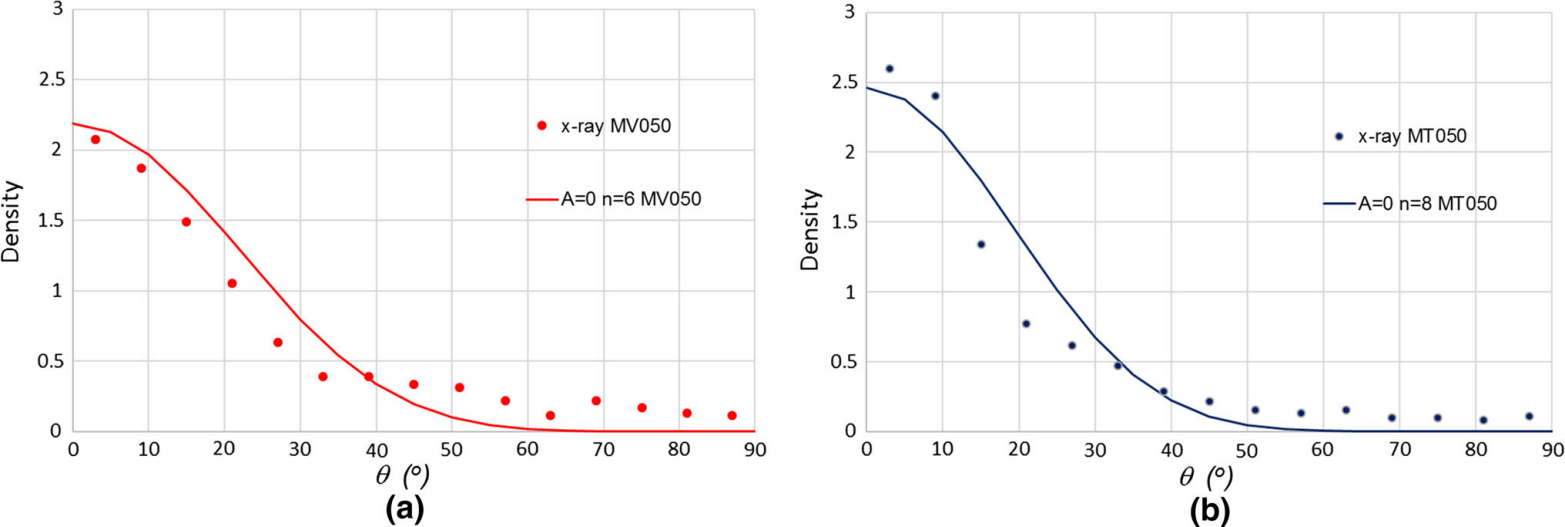
Representation of fibre orientation distributions resulted from X-ray analysis and fibre orientation procedure proposed by Diambra et al. [26] based on the fibre orientation function defined by relation (2): **a** MV050 and **b** MT050 samples; note that $\theta$ is the angle from horizontal

The experimental procedure requires the physical counting of the number of fibres $$N^{H}$$ and $$N^{V}$$ intersecting horizontal ($$A_H $$ circular area) and vertical ($$A_V $$ rectangular area) sections cut through the cylindrical sample, respectively. In this study, $$N^{H}$$ and $$N^{V}$$ are directly provided by the X-ray horizontal and vertical slices as shown as an example in Fig. 13. For one sample, five horizontal sections (avoiding top and bottom sections because of end effects) and six vertical sections are selected and for each section the number of cut fibres intersecting these planes are visually counted. The average number of fibres counted per vertical and horizontal cut sections for both the MT050 and MV050 samples are summarised in Table 4.

The developments by Diambra et al. [26] provide analytical solutions for $N^{H}$ and $N^{V}$ as function of the fibre orientation distribution function, $\rho \left( \theta \right)$:

5 $$\begin{aligned} N^{H}= & {} \frac{4A_H }{\pi ^{2}l_f d_f^2 }\mathop {\int }\nolimits _0^{l_f /2} \left( {\mathop \int \nolimits _{\frac{\pi }{2}-\hbox {arccos}\left( {2b/l_f } \right) }^{\frac{\pi }{2}} \mathop \int \nolimits _0^{2\pi } \rho \left( \theta \right) \hbox {cos}\left( \theta \right) d\alpha d\theta } \right) db \nonumber \\ \end{aligned}$$

6 $$\begin{aligned} N^{V}= & {} \frac{\hbox {16}A_V }{\pi ^{2}l_f d_f^2 }\mathop {\int }\nolimits _0^{l_f /2} \left( {\mathop {\int }\nolimits _0^{\hbox {arccos}\left( {2b/l_f } \right) } \mathop {\int }\nolimits _0^\alpha \rho \left( \theta \right) \hbox {cos}\left( \theta \right) d\alpha d\theta } \right) db \nonumber \\ \end{aligned}$$

where *b* is a variable defining the distance from the cut plane to the centre of a generic fibre.

The parameters *A* and *n* [*B* is directly evaluated using relation (4)] are then adjusted such that the analytical expressions and experimentally measured $N^{V}, N^{H}$ and $N^{V}{/}N^{H}$ ratio values closely match each other. In the process, the parameter *A* has been set equal to zero (a simplified assumption employed also by Diambra et al. [26] and Ibraim et al. [30] meaning that no fibres have a vertical orientation) and only the parameter *n* has been calibrated against the $N^{V}, N^{H}$ and $N^{V}{/}N^{H}$ experimental values. Table 4 also shows the values of the parameters *n* and *B* and the corresponding analytical estimations of $N^{V},N^{H}$ and $N^{V}/N^{H}$ based on relations (5) and (6). A perfect fit between the number of cut fibres from experiment and the analytical model is not always possible as it is necessary for *n* to be an integer in the integration. Note that a limit value $A=1$ corresponds to an isotropic fibre orientation.

Diambra et al. [26] showed that in a sample with fibre orientation distribution described by (2), the probability density function for the distribution of fibre orientation is given by the following function:

7 $$\begin{aligned} f\left( \theta \right) =\rho \left( \theta \right) \hbox {cos}\left( \theta \right) /\bar{\rho } \end{aligned}$$

The probability density function (7) is plotted versus $\theta$ for the determined values of *A*, *n* and *B* parameters (Table 4) for both MT050 (in Fig. 14a) and MV050 (in Fig. 14b) samples to parallel the fibre orientation distribution directly obtained from the X-ray analysis. The fibre orientation distribution resulted from the X-ray analysis is well described by the proposed fibre orientation function (2). Therefore, the analytical function (2) can confidently be used for the description of fibre orientation distribution in granular soil, at least for mixtures formed in laboratory conditions. In addition, the close match between X-ray data and the formulation resulted from the combined experimental and analytical method validate the latter and gives confidence for use in applications where direct assessment of fibre orientation distribution cannot be performed.

## Conclusion

This study illustrates the potential of X-ray tomography technique for studying the internal architecture of fibres embedded in a granular soil composite. The following results can be listed:

- The work presents the development of specific techniques for fibre identification and micromechanical exploration of soil composites.
- The X-ray analysis shows increased porosity in the fibre vicinity—in this case three fibre diameters—thus confirming the assumption of a “stolen void ratio” effect of fibre presence made by the authors in previous studies.
- MT and MV laboratory fabrication methods create anisotropic orientations of fibres with preferential sub-horizontal directions. The sample layering in MT appears to have a limited effect on the fibre orientation distribution.
- The fibre orientation distribution does not seem to be affected by the concentration of fibres, at least for the fibre concentration ranges considered in this study.
- For both fabrication methods, the fibre orientation distribution appears to be axisymmetric with respect to the vertical axis of the sample.
- The combined experimental and analytical method previously developed is tested and successfully validated against X-ray data. Fibre orientation distribution function of the form given by (2) can be used for the description of fibre orientation in laboratory fabricated samples.
- The size of the samples analysed in this study remains representative, and the results appear to match those obtained on larger laboratory samples provided that a scaling procedure of fibre length is applied.
